# Theoretical and practical knowledge of Nursing professionals on indirect blood pressure measurement at a coronary care unit

**DOI:** 10.1590/S1679-45082014AO2984

**Published:** 2014

**Authors:** Juliana Pereira Machado, Eugenia Velludo Veiga, Paulo Alexandre Camargo Ferreira, José Carlos Amado Martins, Ana Carolina Queiroz Godoy Daniel, Amanda dos Santos Oliveira, Patrícia Costa dos Santos da Silva

**Affiliations:** 1 Escola de Enfermagem de Ribeirão Preto, Universidade de São Paulo, Ribeirão Preto, SP, Brazil;; Centro Universitário Barão de Mauá, Ribeirão Preto, SP, Brazil.; 2 Escola de Enfermagem de Ribeirão Preto, Universidade de São Paulo, Ribeirão Preto, SP, Brazil.; 3 Escola Superior de Enfermagem de Coimbra, Coimbra, Portugal.; 4 Hospital Israelita Albert Einstein, São Paulo, SP, Brazil.

**Keywords:** Nursing, team, Blood pressure determination/nursing, Blood pressure determination/methods, Health knowledge, attitudes, practice, Questionnaires

## Abstract

**Objective:**

To determine and to analyze the theoretical and practical knowledge of Nursing professionals on indirect blood pressure measurement.

**Methods:**

This cross-sectional study included 31 professionals of a coronary care unit (86% of the Nursing staff in the unit). Of these, 38.7% of professionals were nurses and 61.3% nurse technicians. A validated questionnaire was used to theoretical evaluation and for practice assessment the auscultatory technique was applied in a simulation environment, under a non-participant observation.

**Results:**

To the theoretical knowledge of the stages of preparation of patient and environment, 12.9% mentioned 5-minute of rest, 48.4% checked calibration, and 29.0% chose adequate cuff width. A total of 64.5% of professionals avoided rounding values, and 22.6% mentioned the 6-month deadline period for the equipment calibration. On average, in practice assessment, 65% of the steps were followed. Lacks in knowledge were primary concerning lack of checking the device calibration and stethoscope, measurement of arm circumference to choose the cuff size, and the record of arm used in blood pressure measurement.

**Conclusion:**

Knowledge was poor and had disparities between theory and practice with evidence of steps taken without proper awareness and lack of consideration of important knowledge during implementation of blood pressure measurement. Educational and operational interventions should be applied systematically with institutional involvement to ensure safe care with reliable values.

## INTRODUCTION

Hypertension is an important public health concern and represents a main risk factor for cardiovascular diseases, which constitutes one of the most onerous disease that causes severe complication and presents sequelae.^(1)^ Only half of hypertensive individuals have well controlled blood pressure (BP),^(2)^ and a great difficult is seen toward adherence to drug therapy among hypertensive patients.^(3)^

Therapeutic approach to individuals with hypertension should be focused on maintenance of BP <140x90mmHg and considerations for the presence of associated risk factors.^(1,4)^

Indirect BP measurement by the auscultatory method is the most widely used^(1)^ method, but it can present errors related to the environment, observer, patient and device itself, however, it is a simple and easy procedure to be conducted.^(5)^ For this reason, hypertension guidelines describing steps for BP measurement reinforce the importance of obtaining reliable values to support adequate diagnosis and treatment of hypertension.^(1,4,6,7)^

Currently, the use of oscillometric device for BP measurement has been increasing gradually at outpatients units, residencies and, mainly, at hospitals.^(6)^ The oscillometric technique reduces errors related to the observer,^(5)^ and is also influenced by steps for patient preparation. This technique contributes for ranges such as the use of inappropriate cuff size,^(6)^ which deserves rigorous accomplishment in preparatory steps for BP measurement.

Among health professionals there are evidence of failures on measuring BP such as cuff-type chosen, patient position, values rounding and inadequate premeasurement rest.^(8,9)^ Nursing professionals, even registered nurses, have poor knowledge regarding BP measurement.^(10-12)^

This study is of great importance to support planning and implementation of actions and promote improvements in practice of indirect BP measurement particularly for the scarcity of studies to support specific educational and operational interventions for safety and guarantee of reliable values to guide interdisciplinary clinical management.

## OBJECTIVE

To determine theoretical and practical knowledge of Nursing professionals in a coronary unit concerning the steps of indirect blood pressure measurement.

## METHODS

This cross-sectional and descriptive study was carried out in the Coronary Unit of Emergency Department of *Hospital das Clínicas da Faculdade de Medicina de Ribeirão Preto da Universidade de São Paulo*, because of the impact of BP in clinical decision management. All professionals working in the unit were included in the study. Those professionals on vacation or those with any special leave permission were excluded.

This study was approved by Ethical and Research Committee of *Escola de Enfermagem de Ribeirão Preto da Universidade de São Paulo*, protocol number 1418/2011, March 13 2012. Each professional was invited by this study’s researcher and, after acceptance, they were informed on risks, benefits and objective of participation. All participants signed the consent form.

The questionnaire used for data collection was created and validated by the same research group of this study (procedures of creation and validation of this questionnaire are about to be published). The questionnaire was composed by 28 questions and included issues concerned indirect BP measurement, according to guidelines.^(1,4,6,13)^ Of these questions, 20 were related to indirect BP measurement. Questions were concerned patient preparation (information that must be checked before measurement); preparation of the environment (ideal conditions for BP measurement); device checking (calibration, selection and choice of adequate cuff, according to patient’s arm, cuff placement and stethoscope); patient’s position (accommodation, position of the dorsum, legs and arms); values obtaining and recording (estimation of systolic by palpation, value record without rounding, presentation in mmHg, interval between the two measures, time between checking and recording, arm used in the measurement); and care with the device (assessment of extensions and connections, functionality and knowledge of deadlines for calibration date).

Each participant had to show their knowledge regarding theory and practice of BP measurement technique. Activities were conducted in a simulation environment in a Nursing office, and participants acted as a nurse or Nursing technician. During simulation the patient, represented by an actor (role play) was called,^(14)^ and asked to seat in a chair in order to measure the BP using the auscultation technique. All simulations were followed by an assistant of our study whose responsibility was only to observe and record the steps in a validated checklist,^(15)^ which is also about to be published and based on Brazilian guidelines.^(1)^

To standardize the scenario, we used an office table with a computer, two chairs, two sphygmomanometers, two stethoscopes, a tape measure and a form for the patients’ medical record. All simulations occurred in the same acclimatized room and the door of the room was kept open in the beginning of the activity. The patient/actor waited in an anteroom and went to the Nursing office after have his/her name called. The patient should be seated, in a chair with legs crossed and arms lying on his/her belly. The patient history considered was standard: he/she denied symptoms within the last 30 minutes of full bladder and food intake, consumption of alcohol, coffee or cigarettes. In addition, the patient reported walking for 10 minutes and get to health service in the exactly moment of his/her consultation for the first visit.

First the professional conducted practice and then responded the theory assessment in order to not be biased by any information provided in the questionnaire.

Results were typed twice and recorded in a designed database (Microsoft Excel^TM^), and then submitted to descriptive statistical analysis using frequency of categorical and dichotomous responses presented in absolute and relative numbers.

## RESULTS

To determine and analyze theoretical and practical knowledge of Nursing professionals related to the steps of BP measurement, the sample of this study was composed by 31 participants with mean age of 33.1 years. A total of 20 participants (64.5%) were women. Among participants there were 12 nurses (38.7%), 19 Nursing auxiliary or technician (61.3%). Of these, 17 (54.9%) professionals had 5 or more years of experience in the area. Of those who were trained on BP measurement after professional education (n=11), 6 (54.5%) mentioned to receive training within at least 2 years.

Results on preparation of patients and environment for indirect BP measurement (Table 1) showed satisfactory theoretical knowledge related to practice of physical exercise before measurement.

**Table 1:** Frequency of correct answers on preparation of patients and environment for blood pressure measurement among Nursing professionals of the coronary unit

Measurement step	Theoretical assessment n (%)	Practical assessment n (%)
Preparation of patient and environment		
Full bladder was checked	23 (74.2)	19 (61.3)
Physical exercises within, 60 minutes before measurement was checked	28 (90.3)	18 (58.1)
Consumption of alcohol within 30 minutes before consultation was checked	5 (16.1)	4 (12.9)
Consumption of coffee/food within 30 minutes before consultation was checked	8 (25.8)	8 (25.8)
Smoking within 30 minutes before consultation was checked	14 (45.2)	11 (35.5)
Resting of at least 5 minutes before measurement was authorized	4 (12.9)	26 (83.9)
A quiet and calm environment was provided	27 (87.1)	14 (45.2)
Patient was informed to refrain from talking while BP was measured	16 (51.6)	1 (3.2)
Patient was quiet during the procedure	6 (19.4)	29 (93.5)
BP: blood pressure.		

Practical knowledge showed a satisfactory result for patient’s position for BP measurement (Table 2), which was a different result from theoretical knowledge. The steps describing care with the device (Table 3) had low frequency both in theoretical and practical simulation, expect concerning cuff placement. The record of values obtained in the indirect BP measurement (Table 4) was the most negatively affected practice, and this practice consisted of recording the arm used for BP measurement as well as non-rounding values.

**Table 2:** Frequency of correct answers on patient’s position for blood pressure measurement among Nursing professionals of the coronary unit

Measurement step	Theoretical assessment n (%)	Practical assessment n (%)
Patient’s position		
Patient’s arm was positioned at heart level	23 (74.2)	26 (83.9)
Patient maintained his/her arm supported on a firm surface	10 (32.3)	28 (90.3)
Patient’s elbow was slightly flexed	4 (12.9)	28 (90.3)
Patient’s hand palm was held up	5 (12.9)	27 (87.1)
Patient was asked to remove some clothes for cuff placement	1 (3.2)	24 (77.4)
Patient kept his/her legs uncrossed	22 (71.0)	24 (77.4)

**Table 3:** Frequency of correct answers on care with device for blood pressure measurement among Nursing professionals of the coronary unit

Measurement step	Theoretical assessment n (%)	Practical assessment n (%)
Care with the device		
Deadline for device calibration date was checked	13 (58.1)	5 (16.1)
Patients’ arm circumference was measured	18 (58.1)	5 (16.1)
Cuff was selected based on arm size	1 (3.2)	8 (25.8)
Cuff was placed without looseness	3 (9.7)	28 (90.3)
Cuff was placed 2-3 cm above the antecubital fossa	14 (45.2)	25 (80.6)
Center of cuff was centered over brachial artery	15 (48.4)	15 (48.4)
Inadequate use of cuff can influence values	30 (96.8)	-

**Table 4:** Frequency of correct answers obtained and recorded concerning values for blood pressure measurement among nursing professionals of the coronary unit

Measurement step	Theoretical assessment n (%)	Practical assessment n (%)
Obtained and recorded values		
Patient waited for 1 minute to next measurement	8 (25.8)	11 (35.5)
Inflated up to 20-30mmHg above estimated systolic blood pressure	18 (58.1)	27 (87.1)
Systolic blood pressure was determined using auscultation of 1st song (Korotkoff phase I)	21 (67.7)	31 (100.0)
Professional recorded blood pressure values without rounding values	9 (29.0)	7 (22.6)
Record of arm used for blood pressure	30 (96.8)	3 (9.7)
Record of blood pressure values in mmHg	30 (96.8)	30 (96.8)
Notes made directly on patient’s chart	-	29 (93.5)

## DISCUSSION

Our study highlighted the poor theoretical and practical knowledge of Nursing professionals on steps of indirect BP measurement. In addition, some steps differed among theory and practice.

Participants’ mean age was similar to that found in a previous study.^(9)^ However, the larger presence of men among professionals was different from other studies that showed men’s presence of 39%,^(9)^ 13%^(12)^ and 8%.^(8)^ Total time that professionals worked in the area was significant and agreed with previous studies (51,8%).^(9)^ Only one third of professionals participated in training on BP measurement after professional education, and this fact shows the few importance that is given to this thematic. Indeed, the revision of conceptions and approaching of procedures must be encouraged^(16,17)^ because the institutional lack of interest, ineffective politics and lack of educative programs can compromise the professional performance in BP measurement.^(18)^ Raising institutional managers’ awareness on the importance of such training become a challenge.^(17)^

On steps comprising the preparation of patients for indirect BP measurement, there was a concern with influence that physical exercises may cause, especially for the increase of systolic BP,^(19)^ that is different from previous results in which this care type was less valuable mainly by Nursing auxiliary and technicians.^(8,20)^

Results of this study showed poor and inadequate professionals’ practical knowledge on consumption of coffee, alcohol and food and smoking by the patient before measurement. This result is a concern because to skip these steps can compromise values obtained of BP. However, these results agree with other Brazilian studies.^(8,21)^ The care given to full bladder, mentioned in a satisfactory way in theory and practice, was slightly higher from results of previous studies involving physicians and nursing of an intensive care unit,^(22)^ and nurses and health community agents.^(20)^ Theoretical knowledge on resting before BP measurement was inadequate, and lower than other study that reported that nursing 27% and Nursing auxiliary 23% had mentioned such care.^(9)^ In simulated care, however, this step was accomplished by most participants, which confirmed the results of Brazilian studies.^(20,23)^ The difference between theory and practice enabled to understand that resting is requested, but little importance is given on this step and on its relation with BP values.

The majority of respondents mentioned the calm environment, rather than a quiet environment, which can be a problem, because noisy interferes in auscultation technique and in relaxing status of the patient. Among studies available, none measured knowledge related to calm environment, but the inclusion of this recommendation in guidelines is essential particularly to promote relaxation for the patient and to avoid interferences in auscultative technique. Privacy is need^(1)^ because patients’ body can be exposed, for the possible disturbance caused to a patient with altered BP and also to avoid tensioned situation. Despite that, in our sample privacy did not receive adequate value.

Related to patient’s position for indirect BP measurement, arm height was mentioned correctly by professionals in both theory and practice environment, which was a higher results compared with a study conducted with nurses^(10)^ and agreed with previous results reported in studies conducted in Brazil^(9,20,23)^ and Canada.^(23)^

The step of keeping the patient’s arm supported on a firm surface was few mentioned in theory, but, during practice, most of professionals accomplished this step – perhaps for the favorable and adequate placement of the room furniture. Another study assessed the practical knowledge on this regard and reported similar results.^(8)^ Such difference confirms the results reported in a Canadian study that identified similar disproportion.^(24)^

Other steps were often done in practice, especially those related to positioning of arm but the same correspondent in theoretical knowledge was not found. Therefore, this finding raises questions on the risks of non-reflexive and automatized practice,^(25)^ especially for bedridden patients and, therefore, raising the need for periodic updates in order to rescue these concepts. In clinical practice, to keep the cuff wrapped around the arm by automaticity technique is common. Theoretical and practical knowledge related to patients keep legs uncrossed were satisfactory and such knowledge were aligned with evidences,^(26)^ such as in data previously reported.^(20,22)^

In theoretical knowledge concerning care with devices, the checking of calibration was little valorized, and this result agrees with a study in which nurses of intensive care unit did not achieve good performance.^(12)^ In practice, calibration was even more ignored, which perhaps can be related to the lack of a habit to use calibrated devices or to check them. These results are in disagreement with a study that reported 88.9% of professionals who answered corrected.^(20)^ In fact, in that study,^(20)^ the question was closed and dichotomic so that it can induce results. Our study used an open question.

In theoretical assessment, most of professionals confirmed inadequate cuff might influence values obtained, which is contrary to the results of a study conducted in São Paulo including countryside health professionals.^(8)^ However, in practice, the steps for brachial circumference measurement and to select the adequate cuff to the arm were not followed, and this fact was confirmed previously in a study with health professionals who often used standard cuff, which is not correlated with brachial circumference.^(8-10)^ As matter of fact, in our sample, professionals were not able to precisely associate the cuff size with BP values, and they conducted measurement without appropriate reflection. However, the unavailability of different cuff sizes needs to be considered,^(27)^ and this can induce the professional to use what is available without reflect on this step relevance. Responsibilities related to this practice deserve reflections, even for the institution^(28)^ at the moment of cuffs purchase.

In practice assessment professionals knowledge on placement of cuff without looseness were satisfactory, and this agrees with other studies,^(20,23)^ however our study had a better performance than a study using a similar approach.^(9)^ Our main concern is that this step was ignored in theoretical knowledge, particularly because cuff adjustment in arm and removal of clothes may compromise the values obtained in BP measurement.^(29)^ Again, this fact raises questions because of routine in practice, careless and lack of adequate reflection showed by professionals.

Our study also found divergences on which value to take as reference for next measurements, in case of difference among arms, when the recommendation to use the highest value is clear.^(1,4,6)^

In theory no consensus was found related to adequate interval between BP measurements, and in practice few professionals respected an interval to conduct the second measurement. Other studies were not specify about time; they only mentioned that an interval was given,^(8)^ or, cited that it was 30 seconds.^(9)^ In the Brazilian guidelines there is no consensus on the adequate interval.^(1)^ However, the great concern is to repeat the measure immediately without let region used for the measurement to reestablish its normal blood flow.

In theory, concerning care of devices, there are understandings of the correct disposal of cuffs with non-adhesive Velcro**™** and with leakages in edges. However, if studies in our area show careless with clinical devices,^(28,30)^ there is no sure if disposal really occur and if there are any criteria for disposal particularly for the difficult of device replacement as reported by participants. Calibration of aneroid devices every six month is little mentioned, which is similar to a previous study; 4.8%.^(9)^ Education activities, institutional programs for devices maintenance^(28)^ along with knowledge on deadlines for revision and calibration date are critical to improve quality in BP measurement.

Our study is consistent and representative because it involved a population of a coronary unit who had an epidemiologic characteristic profile and Nursing team compatible with the region in which the study was conducted. However, we believe our results can not be extrapolated to professional in other levels of care.

## CONCLUSION

This study evidences theoretical and practical knowledge on steps for BP measurement. The professionals were not within standards required by national guidelines, and they did not accomplish several steps required in BP measurement. We also observed lack of specific training for professionals after formal education and important lack of knowledge on calibration, and preventive program for device maintenance as well as criteria for devices and accessories disposal.

Strategies to improve professionals’ knowledge and practice are needed in order to achieve accurate values in blood pressure. There is also the need to invest in the institutional role to promote professional continuous education and management of devices with participation of all institution to establish actions during purchase, maintenance and storage of equipment in order to improve the care delivery as well as provide more safe practices.
