# Supplementary material for: Theoretical and practical knowledge of Nursing professionals on indirect blood pressure measurement at a coronary care unit
Source: Einstein (Sao Paulo). 2014 Jul-Sep;12(3):330–5. doi: 10.1590/S1679-45082014AO2984 (PMC4872945; doi:10.1590/S1679-45082014AO2984)
Supplement: Translation

# Translation: Conhecimento teórico e prático dos profissionais de Enfermagem em unidade coronariana sobre a medida indireta da pressão arterial

**Authors:** Juliana Pereira Machado, Eugenia Velludo Veiga, Paulo Alexandre Camargo Ferreira, José Carlos Amado Martins, Ana Carolina Queiroz Godoy Daniel, Amanda dos Santos Oliveira, Patrícia Costa dos Santos da Silva

**Affiliations:** 1 Escola de Enfermagem de Ribeirão Preto, Universidade de São Paulo, Ribeirão Preto, SP, Brasil;; Centro Universitário Barão de Mauá, Ribeirão Preto, SP, Brasil.; 2 Escola de Enfermagem de Ribeirão Preto, Universidade de São Paulo, Ribeirão Preto, SP, Brasil.; 3 Escola Superior de Enfermagem de Coimbra, Coimbra, Portugal.; 4 Hospital Israelita Albert Einstein, São Paulo, SP, Brasil.

**Keywords:** Equipe de enfermagem, Determinação da pressão arterial/ enfermagem, Determinação da pressão arterial/métodos, Conhecimentos, atitudes e práticas em saúde, Questionários

## Abstract

**Objetivo:**

Determinar e analisar o conhecimento teórico e prático de profissionais de Enfermagem sobre a medida indireta da pressão arterial.

**Métodos:**

Estudo descritivo, transversal, com profissionais de Enfermagem de uma unidade coronariana. Participaram do estudo 31 sujeitos (86% da população), sendo 38,7% enfermeiros e 61,3% técnicos em enfermagem. A avaliação teórica ocorreu por meio de questionário validado, e a prática, com técnica auscultatória, em simulação, sob observação não participativa.

**Resultados:**

Sobre o conhecimento teórico do preparo do cliente e ambiente, 12,9% citaram repouso de 5 minutos, 48,4% conferiram calibração e 29,0% escolheram o manguito correto. Já 64,5% evitaram o arredondamento dos valores, e 22,6% citaram o prazo semestral para a calibração dos aparelhos. Na avaliação prática, em média, 65% das etapas foram cumpridas. Dentre as lacunas desse conhecimento, destacaram-se a ausência de checagem da calibração do aparelho e do estetoscópio, a medida da circunferência braquial para escolher o manguito, e o registro do braço usado na medida. Conclusão: O conhecimento foi insatisfatório, com discrepâncias entre a teoria e a prática, com indícios de etapas cumpridas sem a devida consciência, e conhecimentos importantes negligenciados na execução da medida da pressão arterial. Intervenções educativas e operacionais devem ser sistematicamente aplicadas, com o envolvimento institucional, para garantir segurança da assistência com valores fidedignos.

## INTRODUÇÃO

A hipertensão arterial (HA) é um importante problema de saúde pública, constituindo o principal fator de risco para doenças cardiovasculares (DCV), que, por sua vez, são as mais onerosas e severas, em complicações e sequelas.^(1)^ Somente metade das pessoas hipertensas possui sua pressão arterial (PA) controlada^(2)^ e, em nosso meio, há franca dificuldade de adesão dos hipertensos ao tratamento medicamentoso.^(3)^

A abordagem terapêutica ao hipertenso deve focar na manutenção da PA em valores <140x90mmHg e considerar a presença de fatores de risco associados.^(1,4)^

Para a medida da PA, o método mais utilizado é o indireto, com técnica auscultatória,^(1)^ o qual pode apresentar erros relacionados ao ambiente, ao observador, ao cliente e ao aparelho, mesmo sendo um procedimento simples e fácil de ser realizado.^(5)^ Por isso, diretrizes de HA descreveram as etapas da medida da PA, reforçando sua importância na obtenção de valores fidedignos, que suportem adequadamente o diagnóstico e tratamento.^(1,4,6,7)^

Simultaneamente, os aparelhos oscilométricos para medida da PA vêm gradualmente aumentando em ambulatórios, residências e, principalmente, em hospitais.^(6)^ A técnica oscilométrica reduz erros relacionados ao observador,^(5)^ contudo, é igualmente influenciada por etapas de preparação do cliente, que contribuem para variações, como, por exemplo, o uso de manguito de tamanho inapropriado,^(6)^ merecendo rigor no cumprimento das etapas preparatórias para a medida da PA.

Entre os profissionais de saúde, há evidências de falhas na execução da medida da PA, como na escolha do manguito, na posição do cliente, no arredondamento de valores e no repouso inadequado antes da medida.^(8,9)^ Entre profissionais de Enfermagem, o conhecimento é insatisfatório, inclusive entre enfermeiros.^(10-12)^

Pela escassez de estudos que possam subsidiar a elaboração de intervenções educativas e operacionais específicas, visando à segurança e à garantia dos valores fidedignos para direcionar as condutas clínicas interdisciplinares, entende-se que o presente estudo é de grande importância para embasar o planejamento e a implementação de ações que promovam melhorias da prática da medida indireta da PA.

## OBJETIVO

Determinar o conhecimento teórico e prático de profissionais de Enfermagem de uma unidade coronariana sobre as etapas da medida indireta da pressão arterial.

## MÉTODOS

Estudo descritivo e transversal, conduzido na Unidade Coronariana da Emergência do Hospital das Clínicas da Faculdade de Medicina de Ribeirão Preto da Universidade de São Paulo, tendo em vista a magnitude do gerenciamento da PA nas decisões clínicas nesse setor. A amostra foi composta pela totalidade dos profissionais alocados na unidade, disponíveis em período de trabalho. Excluíram-se os profissionais que estavam em férias ou afastamento.

O projeto foi aprovado pelo Comitê de Ética em Pesquisa da Escola de Enfermagem de Ribeirão Preto da Universidade de São Paulo, sob o protocolo 1418/2011, em 13 de março de 2012. Cada profissional foi convidado pessoalmente pela pesquisadora e, após aceitação, manifestou clareza e ciência dos riscos e benefícios de sua participação, além de assinar espontaneamente o Termo de Consentimento Livre e Esclarecido (TCLE).

A coleta de dados deu-se por meio de questionário elaborado e validado pelo mesmo grupo de pesquisa deste estudo, cujos resultados estão em fase de publicação, com questões relacionadas às etapas da medida indireta da PA, segundo diretrizes.^(1,4,6,13)^ Tal questionário é composto por 28 questões, sendo 20 delas sobre medida indireta da PA. As perguntas versam sobre o preparo do cliente (informações que devem ser checadas antes da medida); preparo do ambiente (condições ideais para a medida da PA); checagem do aparelho (calibração, seleção e escolha do manguito ideal, de acordo com o braço do cliente, colocação do manguito e estetoscópio); posição do cliente (acomodação, posição do dorso, pernas e braços); obtenção e registros dos valores (estimativa da PA sistólica por palpação, registro dos valores sem arredondamento, em mmHg, intervalo entre duas medidas, tempo entre verificação e registro, braço usado na medida); e cuidados com o aparelho (avaliação de extensões e conexões, funcionalidade e conhecimentos sobre prazos de calibração).

Além do conhecimento teórico, cada participante demonstrou a técnica da medida da PA em ambiente de simulação, num consultório de Enfermagem. Essa atividade transcorreu com o participante no papel de enfermeiro ou técnico de Enfermagem, que deveria chamar o cliente, representado por um ator (*role play*),^(14)^ acomodá-lo e executar a medida da PA na posição sentada, pela técnica auscultatória. A simulação era acompanhada por um auxiliar da pesquisa, por observação não participativa, que registrava as etapas executadas num *checklist* validado,^(15)^ em fase de publicação, baseado nas diretrizes brasileiras.^(1)^

Para padronizar o cenário, foram utilizados: uma mesa de escritório com computador, duas cadeiras, dois aparelhos aneroides, dois estetoscópios, uma fita métrica e uma ficha de atendimento para prontuário clínico do cliente. A simulação ocorreu sempre na mesma sala, climatizada, mantendo-se a porta aberta no início da atividade. O cliente/ator aguardava numa antessala e adentrava ao consultório de Enfermagem assim que fosse chamado. Ele deveria sentar-se sempre com as pernas cruzadas e os braços dispostos sobre o colo; seu histórico era padronizado: negava relato, nos últimos 30 minutos, de bexiga cheia ou ingestão de alimentos, álcool, café e fumo; relatava ter caminhado por 10 minutos e ter chegado ao serviço de saúde naquele momento, para sua primeira consulta.

Inicialmente, o participante realizava a demonstração da técnica e, depois, a avaliação teórica, para que não fosse induzido por alguma informação contida no questionário.

Os resultados foram inseridos em banco de dados em Excel^®^ por dupla digitação e submetidos à estatística descritiva, por frequência de respostas categóricas ou dicotômicas, apresentadas em números absolutos e relativos.

## RESULTADOS

Para determinar e analisar o conhecimento teórico e prático dos profissionais de Enfermagem sobre as etapas da medida da PA, a amostra contou com 31 participantes, com média de idade de 33,1 anos, e 64,5% (n=20) do gênero feminino. Em relação à função, 38,7% (n=12) eram enfermeiros e 61,3% (n=19) técnicos ou auxiliares de Enfermagem; 54,9% (n=17) tinham 5 anos ou mais na função atual. Entre os que tiveram treinamentos sobre a medida da PA após sua formação profissional (n=11), 54,5% (n=6) referiram que foi há pelo menos 2 anos.

Os resultados sobre o preparo do cliente e do ambiente para a medida indireta da PA (Tabela 1) mostraram conhecimento teórico satisfatório sobre exercícios físicos praticados antes da medida.

**Tabela 1:** Frequência de respostas corretas sobre o preparo do cliente e ambiente para a medida da pressão arterial, entre profissionais de Enfermagem de uma unidade coronariana

Etapa da medida	Avaliação teórica	Avaliação prática
	n (%)	n (%)
Preparo do cliente e do ambiente		
Checou bexiga cheia	23 (74,2)	19 (61,3)
Checou exercícios físicos 60 minutos antes	28 (90,3)	18 (58,1)
Checou ingestão bebidas alcoólicas 30 minutos antes	5 (16,1)	4 (12,9)
Checou ingestão de café/alimentos 30 minutos antes	8 (25,8)	8 (25,8)
Checou consumo de tabaco 30 minutos antes	14 (45,2)	11 (35,5)
Permitiu repouso de pelo menos 5 minutos	4 (12,9)	26 (83,9)
Proporcionou ambiente calmo e silencioso	27 (87,1)	14 (45,2)
Orientou o cliente para não conversar	16 (51,6)	1 (3,2)
Manteve-se em silêncio durante o procedimento	6 (19,4)	29 (93,5)

Com relação à posição do cliente para a realização da medida da PA (Tabela 2), o conhecimento prático foi satisfatório, diferentemente do teórico. As etapas que descrevem os cuidados com o aparelho (Tabela 3) obtiveram frequência baixa, tanto na teoria quanto na prática em simulação, exceto a colocação do manguito. A obtenção e o registro dos valores de PA obtidos na medida indireta (Tabela 4), que foram as etapas mais prejudicadas na prática, versaram sobre anotar o membro em que a PA foi medida, bem como os valores sem arredondamento.

**Tabela 2:** Frequência de respostas corretas sobre a posição do cliente para a medida da pressão arterial, entre profissionais de Enfermagem de uma unidade coronariana

Etapa da medida	Avaliação teórica	Avaliação prática
	n (%)	n (%)
Posição do cliente		
Posicionou o braço do cliente na altura do coração	23 (74,2)	26 (83,9)
Manteve o braço do cliente apoiado	10 (32,3)	28 (90,3)
Manteve o cotovelo levemente fletido	4 (12,9)	28 (90,3)
Manteve palma da mão voltada para cima	5 (12,9)	27 (87,1)
Removeu roupas para colocar manguito	1 (3,2)	24 (77,4)
Manteve pernas do cliente descruzadas	22 (71,0)	24 (77,4)

**Tabela 3:** Frequência de respostas corretas sobre os cuidados com o aparelho para a medida da pressão arterial, entre profissionais de Enfermagem de uma unidade coronariana

Etapa da medida	Avaliação teórica	Avaliação prática
	n (%)	n (%)
Cuidados com o aparelho		
Checou o prazo de calibração do aparelho	13 (58,1)	5 (16,1)
Mediu a circunferência do braço do cliente	18 (58,1)	5 (16,1)
Selecionou o manguito de tamanho ideal ao braço	1 (3,2)	8 (25,8)
Colocou o manguito sem deixar folgas	3 (9,7)	28 (90,3)
Colocou o manguito 2-3 acima da fossa anticubital	14 (45,2)	25 (80,6)
Centralizou o meio do manguito sobre a artéria braquial	15 (48,4)	15 (48,4)
Concordou que usar manguito inadequado pode influenciar valores	30 (96,8)	-

**Tabela 4:** Frequência de respostas corretas sobre a obtenção e o registro dos valores para a medida da pressão arterial, entre profissionais de Enfermagem de uma unidade coronariana

Etapa da medida	Avaliação teórica	Avaliação prática
	n (%)	n (%)
Obtenção e registro dos valores		
Aguardou 1 minuto para a próxima medida	8 (25,8)	11 (35,5)
Inflou até 20-30mmHg acima da pressão arterial sistólica estimada	18 (58,1)	27 (87,1)
Determinou pressão arterial sistólica na ausculta do primeiro som (fase I Korotkoff)	21 (67,7)	31 (100,0)
Anotou os valores da pressão arterial sem arredondamentos	9 (29,0)	7 (22,6)
Anotou o membro em que foi verificada a pressão arterial	30 (96,8)	3 (9,7)
Registrou valores de pressão arterial, em mmHg	30 (96,8)	30 (96,8)
Realizou anotação diretamente no prontuário	-	29 (93,5)

## DISCUSSÃO

Este estudo evidenciou frágil conhecimento teórico e prático de profissionais de Enfermagem sobre etapas da medida indireta de PA na amostra estudada. Além disso, algumas etapas diferem entre teoria e prática.

A média de idade foi semelhante a de estudo anterior.^(9)^ Entretanto, a maior presença de profissionais homens diferiu de outros estudos, com 39%,^(9)^ 13%^(12)^ e 8%.^(8)^ O tempo de exercício da função foi considerável e concordou com estudo anterior (51,8%).^(9)^ Apenas um terço da amostra participou de treinamentos sobre a medida da PA após sua formação, demonstrando a pouca importância dada a esse tema. De fato, a revisão de conceitos e a abordagem de procedimentos devem ser encorajadas,^(16,17)^ pois o desinteresse institucional, as políticas ineficazes e a ausência de programas educativos podem comprometer o desempenho profissional na medida da PA.^(18)^ Conscientizar gestores institucionais sobre a importância dessa necessidade tornou-se um desafio.^(17)^

Sobre as etapas que compõem o preparo do cliente para a medida indireta da PA, houve a preocupação com a influência que exercícios físicos podem exercer, sobretudo a elevação da PA sistólica,^(19)^ contrapondo-se a resultados anteriores, nos quais esse cuidado foi pouco valorizado, principalmente por auxiliares e técnicos de Enfermagem.^(8,20)^

Os resultados deste estudo mostraram que os conhecimentos práticos sobre a ingestão de café, álcool, alimentos, e sobre o fumo foram muito frágeis e insatisfatórios, e concordam com outros estudos brasileiros,^(8,21)^ o que pode comprometer os valores de PA obtidos. A atenção à bexiga cheia, mencionada de forma satisfatória na teoria e na prática, foi ligeiramente superior a resultados de estudos anteriores, com médicos e a Enfermagem de Unidade de Terapia Intensiva,^(22)^ e com Enfermagem e agentes comunitários de saúde.^(20)^ O conhecimento teórico sobre repouso antes da medida da PA foi insatisfatório, inferior a estudo em que 27% dos enfermeiros e 23% dos auxiliares de Enfermagem citaram tal cuidado.^(9)^ Na prática simulada, entretanto, essa etapa foi cumprida pela maioria, confirmando estudos brasileiros.^(20,23)^ A discrepância entre teoria e prática permite deduzir que essa etapa é executada sem a devida noção de sua importância em relação aos valores de PA.

A menção sobre o ambiente calmo esteve na maioria das respostas; diferentemente do silêncio, o que pode ser problemático, pois ruídos interferem na técnica auscultatória e no estado de relaxamento do cliente. Dentre os estudos disponíveis, nenhum mensurou o conhecimento sobre essa etapa especificamente, contudo, ressalta-se que a recomendação presente em diretrizes é essencial, sobretudo para promover o estado de relaxamento do cliente e não interferir na técnica auscultatória. A privacidade é necessária^(1)^ pela eventual exposição dos membros, pelo incômodo do cliente com PA alterada e para evitar situação de tensão, porém, nesta amostra, ela foi desvalorizada.

Com relação à posição do cliente para a medida indireta da PA, a altura do braço foi citada corretamente na teoria e prática, sobrepondo-se a estudo com enfermeiros^(10)^ e concordando com resultados anteriores do Brasil^(9,20,23)^ e do Canadá.^(23)^

Manter o braço apoiado foi especificamente uma etapa pouco mencionada na teoria, mas, na prática, a maioria a cumpriu − talvez favorecidos pela disposição adequada do mobiliário. Estudos que avaliaram o conhecimento prático obtiveram resultados semelhantes.^(8)^ Tal discrepância confirma estudo canadense, que obteve igual desproporção.^(24)^

Outras etapas foram muito executadas na prática, sobretudo aquelas relacionadas ao posicionamento do braço, sem o conhecimento teórico correspondente. Tais achados evocam questionamentos sobre o risco da prática não reflexiva e automatizada,^(25)^ sobretudo nos clientes acamados, e levanta necessidade de atualizações periódicas, para resgatar esses conceitos. Na prática clínica, é comum manter continuamente o manguito envolto ao braço pela automaticidade da técnica. Com relação a manter as pernas do cliente descruzadas, o conhecimento teórico e prático foi satisfatório, e mostrou-se alinhado às evidências,^(26)^ a exemplo de dados obtidos anteriormente.^(20,22)^

Sobre os cuidados com os aparelhos, no conhecimento teórico, a checagem da calibração foi pouco valorizada, fato que concorda com estudo em que enfermeiros de terapia intensiva não tiveram bom aproveitamento.^(12)^ A prática foi ainda mais negligenciada, o que talvez possa estar relacionado à falta do hábito de usar dispositivos calibrados ou realizar checagem. Esses resultados discordam de estudo em que 88,9% dos profissionais acertaram essa resposta.^(20)^ Vale dizer que, no referido estudo,^(20)^ a questão era fechada e dicotômica, o que pode ter induzido a tais resultados. Em nosso estudo, a questão era aberta.

Na avaliação teórica, a maioria afirmou que o manguito inadequado pode influenciar os valores obtidos, contrariando dados de estudo entre profissionais de saúde do interior paulista.^(8)^ Adversamente, na prática, as etapas de medir a circunferência braquial e selecionar o manguito adequado ao braço foram muito negligenciadas, o que confirmou dados anteriores, em que profissionais de saúde utilizaram preferencialmente manguito padrão e não correlacionaram com circunferência braquial.^(8-10)^ De fato, nesta amostra, os profissionais não conseguiram associar o tamanho do manguito com a precisão dos valores de PA e executam a medida sem a devida reflexão. Contudo, é necessário considerar a indisponibilidade de diferentes tamanhos de manguitos,^(27)^ o que pode tendenciosamente, induzir o profissional a usar o que tem disponível, sem refletir sobre a relevância dessa etapa. As responsabilidades intrínsecas a essa prática merecem reflexões, inclusive institucionais,^(28)^ no momento da aquisição desses manguitos.

Sobre a colocação do manguito sem folgas, na avaliação prática, o conhecimento foi satisfatório, confirmando outros estudos,^(20,23)^ e teve melhor desempenho, comparando-se a estudo semelhante.^(9)^ Preocupa, porém, o fato dessa etapa ter sido ignorada no conhecimento teórico, pois o ajuste ao braço e a remoção de roupas podem comprometer os valores obtidos.^(29)^ Novamente, esse fato suscita questionamentos sobre a prática rotineira, descuidada e sem a devida reflexão.

O estudo evidenciou também divergências sobre qual valor tomar como referência para as próximas medidas, no caso de diferença entre os braços, quando é clara a recomendação é utilizar o maior valor.^(1,4,6)^

Sobre o intervalo para outra medida de PA, não houve consenso na teoria e, na avaliação prática, ele foi cumprido por poucos. Outros estudos não foram específicos com relação ao tempo; somente citaram que houve intervalo,^(8)^ ou, então, que este foi de 30 segundos.^(9)^ É bem verdade que as diretrizes brasileiras informam não haver consenso.^(1)^ O mais preocupante é, contudo, repetir a medida imediatamente, sem que a região comprimida possa restabelecer o fluxo sanguíneo normal.

Nos cuidados com aparelhos, na teoria, há entendimento correto de descartar manguitos com Velcro^®^ não aderente e extensões com vazamentos. Ora, se estudos em nosso meio demonstram descuido com aparelhos na clínica,^(28,30)^ questiona-se se os descartes acontecem de fato e se existe algum critério para que isso ocorra diante da dificuldade de reposição verbalizada pelos participantes. A calibração semestral dos aparelhos aneroides é citada minimamente, tal como em estudo anterior, com 4,8%.^(9)^ Além das atividades educativas, programas institucionais de manutenção de equipamentos,^(28)^ aliados ao conhecimento sobre prazos de revisão e calibração, são imprescindíveis para a melhoria da qualidade da medida da PA.

Os dados são consistentes e representativos para a amostra estudada, pois esta compreende a população de uma unidade coronariana com perfil epidemiológico característico e equipe de Enfermagem compatível com a região na qual está inserida. Entretanto, acredita-se que não possam ser generalizados para profissionais de outros níveis de atenção.

## CONCLUSÃO

Esse estudo evidenciou que o conhecimento teórico e prático sobre as etapas da medida da pressão arterial, nesta amostra, está aquém das diretrizes, e muitas etapas da medida não foram cumpridas. Também se evidenciaram escassez de treinamentos específicos após a formação e grande desconhecimento sobre calibração, ausência de programas de manutenção preventiva e critérios de descarte de aparelhos e acessórios.

Estratégias de melhoria do conhecimento e da prática são necessárias para a obtenção de valores de pressão arterial fidedignos, assim como é importante investir no papel das instituições na atualização profissional e na gestão de equipamentos, com ações institucionais participativas, desde a aquisição, a manutenção e a guarda de equipamentos, para melhorar a assistência prestada à medida que proporciona uma prática mais segura.
